# Using modified Fenn diagrams to assess ventilatory acclimatization during ascent to high altitude: Effect of acetazolamide

**DOI:** 10.1113/EP091748

**Published:** 2024-05-15

**Authors:** Rodion Isakovich, Valerie C. Cates, Brandon A. Pentz, Jordan D. Bird, Emily R. Vanden Berg, Emily M. de Freitas, Cassandra E. Nysten, Jack K. Leacy, Ken D. O'Halloran, Thomas D. Brutsaert, Mingma T. Sherpa, Trevor A. Day

**Affiliations:** ^1^ Department of Biology, Faculty of Science and Technology Mount Royal University Calgary Alberta Canada; ^2^ Department of Physiology, School of Medicine, College of Medicine & Health University Cork College Cork Ireland; ^3^ Department of Exercise Science Syracuse University Syracuse New York USA; ^4^ Kunde Hospital Khunde, Solukhumbu Nepal

**Keywords:** acclimatization, acetazolamide, acute mountain sickness, high altitude ascent, hypoxic ventilatory response

## Abstract

**Abstract:**

High altitude (HA) ascent imposes systemic hypoxia and associated risk of acute mountain sickness. Acute hypoxia elicits a hypoxic ventilatory response (HVR), which is augmented with chronic HA exposure (i.e., ventilatory acclimatization; VA). However, laboratory‐based HVR tests lack portability and feasibility in field studies. As an alternative, we aimed to characterize area under the curve (AUC) calculations on Fenn diagrams, modified by plotting portable measurements of end‐tidal carbon dioxide ($P_{\mathrm{ETC}O_{2}}$) against peripheral oxygen saturation ($S_{pO_{2}}$) to characterize and quantify VA during incremental ascent to HA (*n* = 46). Secondarily, these participants were compared with a separate group following the identical ascent profile whilst self‐administering a prophylactic oral dose of acetazolamide (Az; 125 mg BID; *n* = 20) during ascent. First, morning $P_{\mathrm{ETC}O_{2}}$ and $S_{pO_{2}}$ measurements were collected on 46 acetazolamide‐free (NAz) lowland participants during an incremental ascent over 10 days to 5160 m in the Nepal Himalaya. AUC was calculated from individually constructed Fenn diagrams, with a trichotomized split on ranked values characterizing the smallest, medium, and largest magnitudes of AUC, representing high (*n* = 15), moderate (*n* = 16), and low (*n* = 15) degrees of acclimatization. After characterizing the range of response magnitudes, we further demonstrated that AUC magnitudes were significantly smaller in the Az group compared to the NAz group (*P *= 0.0021), suggesting improved VA. These results suggest that calculating AUC on modified Fenn diagrams has utility in assessing VA in large groups of trekkers during incremental ascent to HA, due to the associated portability and congruency with known physiology, although this novel analytical method requires further validation in controlled experiments.

**Highlights:**

- **What is the central question of this study?**
  What are the characteristics of a novel methodological approach to assess ventilatory acclimatization (VA) with incremental ascent to high altitude (HA)?
- **What is the main finding and its importance?**
  Area under the curve (AUC) magnitudes calculated from modified Fenn diagrams were significantly smaller in trekkers taking an oral prophylactic dose of acetazolamide compared to an acetazolamide‐free group, suggesting improved VA. During incremental HA ascent, quantifying AUC using modified Fenn diagrams is feasible to assess VA in large groups of trekkers with ascent, although this novel analytical method requires further validation in controlled experiments.

## 1 INTRODUCTION

Incremental ascent to high altitude (HA) progressively imposes hypoxic stress on the body and leads to systemic hypoxia (e.g., West, 2012). As the pressure of atmospheric oxygen decreases with ascent, the partial pressure of oxygen in arterial blood is reduced ($P_{aO_{2}}$; hypoxaemia), which is then sensed by peripheral chemoreceptors (i.e., carotid bodies; e.g., Teppema & Dahan, 2010). In response to hypoxaemia, a peripherally mediated hypoxic ventilatory response (HVR) is elicited, whereby ventilation is increased, partially restoring oxygenation (e.g., Sato et al., 1992). However, hyperventilation decreases the partial pressure of carbon dioxide ($P_{\mathrm{aC}O_{2}}$) leading acutely to respiratory alkalosis, blunting both central and peripheral chemoreceptors, inhibiting the HVR (Sato et al., 1992). Within hours, the kidneys begin excreting bicarbonate ions (HCO_3_^−^) and retain hydrogen ions (H^+^) to decrease blood pH (e.g., Ge et al., 2006), partially correcting acid–base balance (i.e., metabolic acidosis), gradually intensifying the HVR with renal acclimatization to HA (e.g., Bird, Leacy et al. 2021; Krapf et al., 1991; Zouboules et al., 2018). In addition, after days to weeks of hypoxic exposure, HVR sensitivity increases due to ventilatory acclimatization to hypoxia (VA; Ainslie et al., 2013; Powell et al., 2000; Sato et al., 1992; Schoene et al., 1990). During this process, sensitization to low oxygen occurs through (a) increased sensitivity of carotid body type I glomus cells and (b) plasticity within the nucleus tractus solitarius of the brainstem to afferent signals from peripheral chemoreceptors (Dwinell & Powell, 1999; Moya et al., 2020; Powell et al., 2000; Sato et al., 1992; Wang et al., 2008). As a result, VA increases resting ventilation, increasing $P_{aO_{2}}$ and partly mitigating the effects of systemic hypoxia (Ivy & Scott, 2017). The importance of VA has been extensively documented, with observations of a protective effect of VA on $P_{aO_{2}}$ and oxygen saturation ($S_{O_{2}}$; Bernardi, 2006; Sato et al., 1992; Schoene et al., 1984). In addition, self‐reported acute mountain sickness (AMS) symptoms may be recorded during ascent, as AMS often manifests in unacclimatized mountaineers and trekkers at altitudes above 2500 m, with symptoms ameliorating as individuals acclimatize (Chen et al., 2008). These symptoms may include headache, gastrointestinal problems, fatigue, dizziness, sleep disturbances and negative effects on activity performance (Chen et al., 2008; Roach et al., 2018). Particularly with more rapid ascent profiles and/or higher absolute altitudes, the incidence and severity of altitude illness is higher, but with appreciable variability between individuals (Schneider et al., 2002).

Although renal compensation occurs with ascent (e.g., Bird, Leacy et al. 2021; Zouboules et al., 2018), oral acetazolamide (e.g., Diamox) administration is commonly used to aid in enhancing VA, and preventing or treating altitude‐related illnesses, including AMS (e.g., Luks et al., 2019; Swenson et al., 2016). Acetazolamide is a carbonic anhydrase inhibitor, which enhances the renally mediated compensatory metabolic acidosis response to respiratory alkalosis during ascent by blocking extracellular and intracellular carbonic anhydrase in renal tubules. Additional HCO_3_^−^ is not reabsorbed from the filtrate into the blood, and systemic H^+^ is subsequently retained (Chakraborti et al., 1985; Hamm et al., 2015; Krapf et al., 1991), creating a further metabolic acidosis. Acetazolamide‐induced metabolic acidosis aids in VA by stimulating both central and peripheral respiratory chemoreceptors, and thus increasing ventilation and subsequent oxygenation with ascent (e.g., Swenson et al., 2016).

Transient or steady‐state hypoxia tests are the most common methods to assess HVR in a laboratory setting (e.g., Steinback & Poulin, 2007). However, because they aim to isolate peripheral from central chemoreceptors, these HVR tests overlook the potential interaction between the two chemoreceptor compartments (Powell et al., 2000; Wilson & Teppema, 2016). In addition, the sympathetic, cardiovascular and cerebrovascular responses to acute hypoxia may confound these measures during steady‐state isocapnic tests (Steinback & Poulin, 2008), while steady‐state poikilocapnic conditions decrease the activation of central chemoreceptors, also confounding HVR measurements (Steinback & Poulin, 2007). Furthermore, it is challenging to transport the necessary equipment for HA fieldwork studies as it may be expensive, heavy and fragile, and some procedures that assess VA may also lack feasibility because they require the application of additional hypoxic exposure in environments that already expose the participant to hypobaric hypoxia. Therefore, novel methods that assess VA during HA ascent are necessary to accurately quantify VA while minimizing costs and improving feasibility, particularly for larger groups ascending to HA.

**FIGURE 1:**
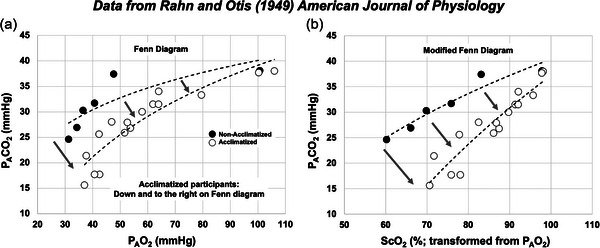
Original and modified Fenn diagrams from Rahn and Otis (1949). (a) Original data from Rahn and Otis (1949) using $P_{\mathrm{AC}O_{2}}$ plotted against $P_{AO_{2}}$ in both unacclimatized (filled circles) and acclimatized participants (open circles). (b) Original data from Rahn and Otis (1949), but with calculated oxygen saturation ($S_{cO_{2}}$; %) calculated using the Severinghaus transform (Severinghuas, 1979) from $P_{AO_{2}}$. In both (a) and (b), note that participants that are acclimatized have a Fenn diagram that is shifted down and to the right, owing to improved oxygenation due to increases in ventilation (and concomitant decreases in CO_2_). These demonstrations led to the hypothesis of smaller area under the curve for more acclimatized participants, illustrated in Figure 2.

In their 1949 study, Rahn and Otis (1949) assessed VA by exposing participants to progressively hypoxic environments in a hypobaric chamber, while measuring alveolar partial pressure of oxygen ($P_{AO_{2}}$) and carbon dioxide ($P_{\mathrm{AC}O_{2}}$). After plotting $P_{AO_{2}}$ and $P_{\mathrm{AC}O_{2}}$ values against each other on a Fenn diagram (Fenn et al., 1946), they observed that acclimatized participants were more sensitive to progressive hypoxia than unacclimatized, with a greater hyperventilation response and resulting hypocapnia. These ventilatory responses reduced relative $P_{\mathrm{AC}O_{2}}$ and elevated $P_{AO_{2}}$, resulting in a downward and rightward shift on the Fenn diagram, compared to unacclimatized participants acutely exposed (Rahn & Otis, 1949; see Figure 1a). This finding demonstrates that acclimatized participants can maintain higher oxygenation at increasing simulated altitudes, while maintaining a lower CO_2_ due to the augmented ventilatory response, illustrating VA. However, this technique has not been utilized to assess VA during HA ascent in individual participants using portable devices and measures ($P_{\mathrm{ETC}O_{2}}$ and $S_{pO_{2}}$), and normative descriptions are not available for individual participants to characterize varying degrees of acclimatization. In addition, assessing VA using modified Fenn diagrams in participants using a prophylactic oral dose of acetazolamide (Az) compared to those that are acetazolamide‐free (NAz) has not been characterized.

**FIGURE 2:**
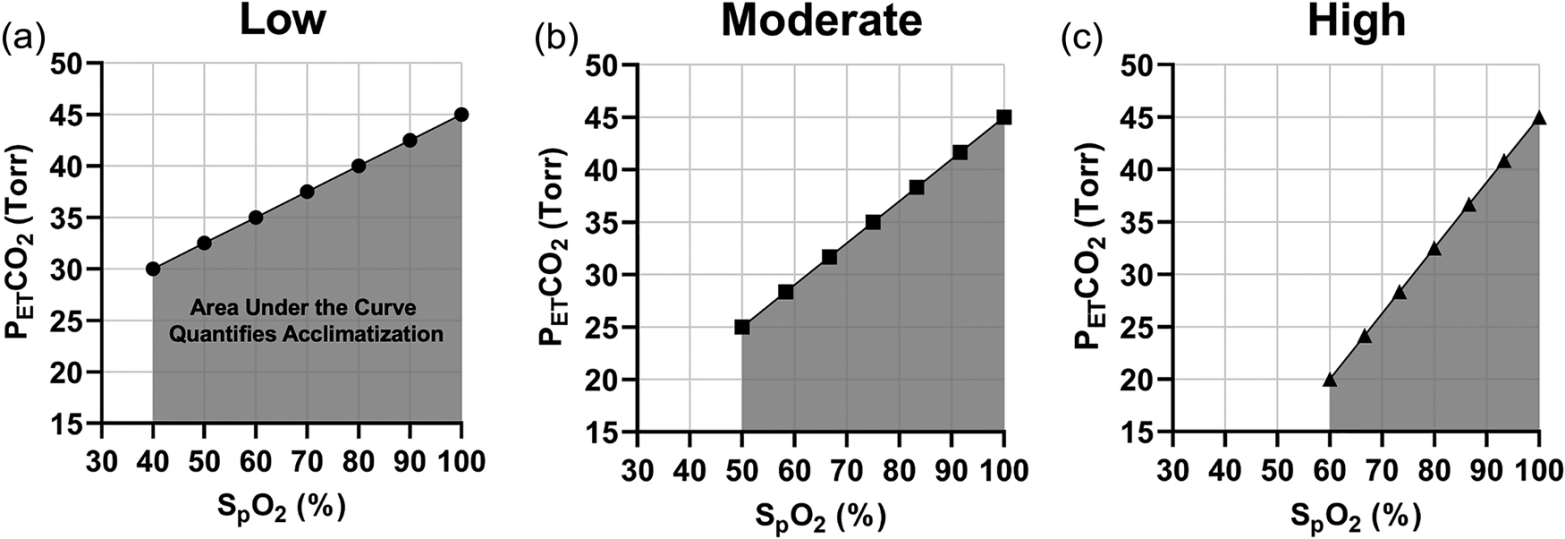
Predicted modified Fenn diagrams for least, moderately and highly acclimatized participants with their respective area under the curve (AUC). End‐tidal partial pressure of CO_2_ ($P_{\mathrm{ETC}O_{2}}$; Torr) was plotted against peripheral oxygen saturation ($SpO_{2}$; %). The least acclimatized participants are predicted to have the largest AUC (Low; a) because of a less prominent hyperventilatory response (HVR), resulting in higher $P_{\mathrm{ETC}O_{2}}$ levels. Moderately acclimatized participants (b) are predicted have a decreased AUC (compared to Low) because of increased HVR, while the smallest AUC is predicted in most‐acclimatized participants (c) due to a more robust HVR, protecting oxygenation and increasing elimination of CO_2_.

In this methodological study, we aimed to characterize a novel analytical method to assess and quantify VA, namely the area under the curve (AUC) on modified Fenn diagrams (i.e., using portable $P_{\mathrm{ETC}O_{2}}$ and $S_{pO_{2}}$ measures), to quantify the range of VA magnitudes in a large group of acetazolamide‐free (NAz) trekkers during an incremental HA ascent profile to 5160 m. Second, we aimed to compare AUC between the NAz group and a separate group following the identical ascent profile but taking a prophylactic oral dose of acetazolamide (Az; 125 mg BID) during ascent. We hypothesized that smaller AUC magnitude on individual Fenn diagrams would represent greater VA (see Figure 2), whereby those participants taking a daily oral prophylactic dose of acetazolamide (Az) with ascent would have a smaller AUC than those that were acetazolamide‐free (NAz).

## 2 METHODS

### 2.1 Ethical approval

This study abided by the Canadian Government Tri‐Council policy on research ethics with human participants (TCPS2) and the *Declaration of Helsinki*, except for registration in a data base. Ethical approval was received in advance through Mount Royal University Human Research Ethics Board (Protocols 2015‐26b and 100012) and was harmonized with the Nepal Health Research Council (Protocols 96‐2015 and 109‐2017).

### 2.2 Participant recruitment

Data collection for the current methodological study took place in the context of several large research expeditions to altitude in the Nepal Himalaya. Prior to voluntary participation in the study, participants were recruited via verbal communication, and each provided verbal and written, informed and ongoing consent to undergo repeated measurements before and during ascent. Participants were all non‐smokers and had no self‐reported history of neurological, cardiovascular, respiratory or metabolic illnesses, nor were they taking any related medications, aside from hormonal birth control, unless they were using acetazolamide (see section below regarding dosage of acetazolamide in the Az group). Due to their participation in an organized and guided expeditions with pre‐determined dates, ovarian cycle in female participants could not be a criterion for inclusion/exclusion in this study, nor was it tracked or controlled for. However, previous reports demonstrated that cycling ovarian hormones do not affect central or peripheral chemoreflex magnitude (Macnutt et al., 2012), but intrinsic sex differences are equivocal. Furthermore, assessing potential sex differences were not planned a priori, but we subsequently assessed this *post hoc*. All participants abstained from alcohol and exercise for at least 12 h prior to measurements, which were all obtained on rest days (i.e., no trekking) following one night at the measurement altitude. At no time was any participant included in the NAz group taking acetazolamide or corticosteroids for the prevention or treatment of altitude‐related illnesses.

Although there is some overlap of ancillary data with previous publications from our group (Bird, Kalker et al. 2021; Bruce et al., 2018; Cates et al., 2022; Holmström et al., 2021; Lafave et al., 2019; Leacy et al., 2021; Zouboules et al., 2018), the comparison of AUC on modified Fenn diagrams during ascent, as well as the comparison between NAz and Az groups, is novel.

### 2.3 Experimental protocols

#### 2.3.1 Experimental approach

The following experimental approach has two distinct components. In Part A, we fully characterize a novel methodological approach to quantify ventilatory acclimatization using area under the curve (AUC) on modified Fenn diagrams during incremental ascent to HA in a large group of acetazolamide‐free trekkers. In Part B, we compare these values from Part A to those from a separate group following an identical ascent profile, but taking a prophylactic dose of acetazolamide (Az).

#### 2.3.2 Ascent profile

All participants completed an identical ascent profile from 1400 m to 5160 m over 10 days, with final measurements made on a rest day on day 10 at 5160 m. Baseline measurements before ascent were performed 2–3 days after arrival in Kathmandu (1400 m). All participants then flew together as a group from 1400 m to 2840 m (Lukla airport) for the first trekking day to 2840 m (Monjo). They arrived at 3440 m (Namche) on day 2 and spent a rest day there (day 3). Participants then ascended to 3820 m (Debuche) on day 4 and stayed for a rest day (day 5). They then ascended to 4240 m (Pheriche) on day 6, stayed for a rest day (day 7), and then ascended to 4910 m (Lobuche) for one night before measurements were made on the morning of day 9. Finally, participants reached 5160 m (Gorak Shep) on day 10 and spent one night there before the last measurements on the morning of day 10.

Atmospheric pressure (*P*_ATM_) at each altitude was not measured directly. To illustrate the incremental HA stressor, *P*_ATM_ was calculated using the ICAO standard atmosphere calculation. This calculation was used to illustrate the stimulus *P*_ATM_ and partial pressure of inspired oxygen ($P_{IO_{2}}$) with ascent (Table 1).

**TABLE 1:** Mean changes in ancillary variables during HA ascent over 10 days.

Variable	1400 m Day 0	2840 m Day 2	3440 m Day 3	3820 m Day 5	4240 m Day 7	4910 m Day 9	5160 m Day 10	*P*
*P*_ATM_ (mmHg)	642	537	497	473	448	410	397	NA
$P_{IO_{2}}$ (mmHg)	125	103	95	90	84	76	74	NA
$S_{pO_{2}}$ (%; mean ± SD)								
All (*n* = 46)	96.26 ± 1.27	93.26 ± 2.42^a^	91.28 ± 3.41^a^,^b^	90.07 ± 3.25^a^	88.30 ± 4.08^a,b^	83.63 ± 4.08^a,b^	78.93 ± 4.83^a,b^	<0.0001
Low (*n* = 15)	96.33 ± 1.11	93.13 ± 2.00^a^	91.27 ± 3.81^a^	88.73 ± 3.41^a^	86.67 ± 2.16^a^	82.13 ± 3.78^a,b^	73.80 ± 2.68^a,b^	<0.0001
Moderate (*n* = 16)	95.81 ± 1.42	92.63 ± 2.94^a^	89.88 ± 3.67^a^	89.25 ± 3.34^a^	88.00 ± 2.76^a^	81.94 ± 3.15^a,b^	79.00 ± 2.58^a^	<0.0001
High (*n* = 15)	96.67 ± 1.18^a^	94.07 ± 2.09^a^	92.80 ± 1.93^a^	92.27 ± 1.58^a^	90.27 ± 2.31^a^	86.93 ± 3.37^a^	84.00 ± 2.24^a^	<0.0001
$P_{\mathrm{ETC}O_{2}}$ (Torr; mean ± SD)								
All (*n* = 46)	31.91 ± 4.38^a^	30.00 ± 3.34^a^	29.40 ± 3.07^a^	27.88 ± 3.13^a,b^	26.95 ± 2.50^a^	27.37 ± 2.33^a^	26.15 ± 2.10^a,b^	<0.0001
Low (*n* = 15)	33.41 ± 4.54^a^	30.57 ± 4.05^a^	29.87 ± 3.58^a^	28.97 ± 3.28^a^	27.40 ± 2.58^a^	28.27 ± 1.64^a^	27.18 ± 1.88^a,b^	<0.0001
Moderate (*n* = 16)	32.16 ± 4.96	30.44 ± 2.78	30.28 ± 2.64	28.41 ± 3.30^b^	26.85 ± 2.83^a^	27.05 ± 1.63^a^	25.61 ± 2.37^a^	<0.0001
High (*n* = 15)	30.14 ± 2.99	28.97 ± 3.07	28.00 ± 2.59	26.24 ± 2.11^a^	26.60 ± 2.12^a^	26.80 ± 3.25	25.71 ± 1.72^a^	0.0001
Daily AMS Score [median(range)]								
All (*n* = 46)	0 (0–1)	0 (0–3)	0 (0–3)	0 (0–3)	0 (0–3)	1 (0–3)^a^	1 (0–6)^a^	<0.0001
Low (*n* = 15)	0 (0–1)	0 (0–1)	0 (0–3)	0 (0–2)	0 (0–2)	1 (0–3)	1 (0–3)^a^	0.0005
Moderate (*n* = 16)	0 (0)	0 (0–1)	0 (0–2)	0 (0–2)	0 (0–3)	1 (0–2)^a^	1.5 (0–6)^a^	<0.0001
High (*n* = 15)	0 (0–1)	0 (0–3)	1 (0–3)	0 (0–3)	0 (0–2)	1 (0–3)	1 (0–4)^a^	0.0002

Data shown for all the participants (*n* = 46), which were divided into groups of low (*n* = 15), moderate (*n* = 16) and high (*n* = 15) degree of acclimatization. ^a^Significantly different from measurements attained at 1400 m, *P *< 0.05. ^b^Significantly different from the preceding attitude, *P *< 0.05. Abbreviations: AMS, acute mountain sickness; *P*_ATM_, atmospheric pressure; $P_{\mathrm{ETC}O_{2}}$, partial pressure of end‐tidal carbon dioxide; $P_{IO_{2}}$, partial pressure of inspired oxygen; $S_{pO_{2}}$, peripheral oxygen saturation. NA, Not applicable.

#### 2.3.3 Daily cardiorespiratory measurements

To characterize physiological responses to incremental ascent to altitude, resting physiological measurements were obtained in the morning between 06.00 and 09.00 h local time following one night sleep at each of the following altitudes: 1400 m (day 0), 2840 m (day 2), 3440 m (day 3), 3820 m (day 5), 4240 m (day 7), 4910 m (day 9) and 5160 m (day 10). All physiological measures were obtained at rest in a seated position following >2‐min rest with eyes closed and white noise played through headphones to limit distraction. Mainstream $P_{\mathrm{ETC}O_{2}}$ (in mmHg; atmospheric pressure adjusted), measured using a portable capnograph (EMMA, Masimo, Danderyd, Sweden) and a personal mouthpiece and nose clip, was obtained from a running average after steady‐state was achieved. A finger pulse oximeter (Masimo Pronto, Masimo Canada, Vancouver, BC, Canada) was used to measure $S_{pO_{2}}$ (as a percentage) by placing it on the left middle finger. Self‐reported AMS scores were then obtained using the updated Lake Louise Questionnaire (Roach et al., 2018). Individual data for $P_{\mathrm{ETC}O_{2}}$, $S_{pO_{2}}$ and AMS scores were all documented by hand, and entered into digital spreadsheets. For consistency in comparison, all baseline values were obtained at 1400 m, and the same data collection protocol was applied for all the participants with ascent.

**FIGURE 3:**
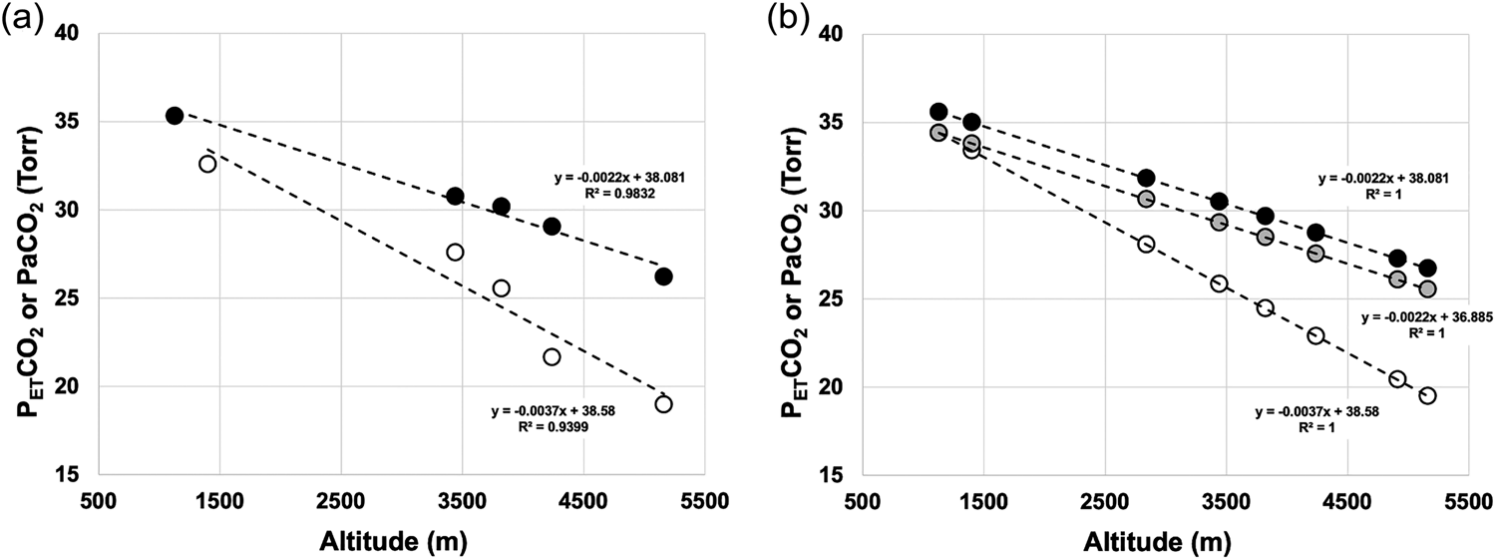
Correction model for $P_{\mathrm{ETC}O_{2}}$ from portable capnograph against $P_{\mathrm{aC}O_{2}}$ from arterial blood draws during incremental ascent to 5160 m. (a) Comparison between trends in $P_{\mathrm{ETC}O_{2}}$ versus $P_{\mathrm{aC}O_{2}}$ during incremental ascent from 1400 m to 5160 m, illustrating divergence with ascent. (b) Linear regression correction functions, correcting $P_{\mathrm{ETC}O_{2}}$ to within a consistent ET‐a difference with incremental ascent. Black circles in (a) and (b), $P_{\mathrm{aC}O_{2}}$ values with ascent. White circles in (a) and (b), $P_{\mathrm{ETC}O_{2}}$ values with ascent. Grey circles in (b), corrected $P_{\mathrm{ETC}O_{2}}$ values. Linear regression formula and *R* ^2^ values for each set of measures reported on graphs. All raw data from Zouboules et al. (2018) (*n* = 22; see Methods). Specifically, to correct $P_{\mathrm{ETC}O_{2}}$ values with ascent, the following values were added to $P_{\mathrm{ETC}O_{2}}$ values obtained from the portable capnograph, within‐individual: 1400 m, +0.405 Torr; 2840 m, +2.565 Torr; 3440 m, +3.465 Torr; 3820 m, +4.035 Torr; 4240 m, +4.665 Torr; 4910 m, +5.67 Torr; 5160 m, +6.045 Torr.

The portable capnograph utilized for $P_{\mathrm{ETC}O_{2}}$ measures is rated for accuracy to an atmospheric pressure equivalent to approximately *P*_ATM_ of ∼525 mmHg (∼3200 m). In a previous study, we noted that (a) $P_{\mathrm{ETC}O_{2}}$ using this model of capnograph underestimated $P_{\mathrm{aC}O_{2}}$ with ascent and (b) the underestimation of $P_{\mathrm{aC}O_{2}}$ by $P_{\mathrm{ETC}O_{2}}$ was exaggerated with ascent to 5160 m following an identical ascent profile (i.e., diverged with ascent; see Zouboules et al., 2018). Specifically, we found (a) the $P_{\mathrm{ETC}O_{2}}$− $P_{\mathrm{aC}O_{2}}$ difference at 1130 m/L, 400 m was −1.2 Torr and (b) the $P_{\mathrm{aC}O_{2}}$ slope with ascent was *y* = −0.0022*x* + 38.081 (*R* ^2^ = 0.98), and the $P_{\mathrm{ETC}O_{2}}$ slope was *y* = −0.0037*x* + 38.58 (*R* ^2^ = 0.94). Accordingly, we corrected the $P_{\mathrm{ETC}O_{2}}$ slope such that the $P_{\mathrm{ETC}O_{2}}$− $P_{\mathrm{aC}O_{2}}$ difference was equivalent with ascent to *y* = −0.0022*x* + 36.885, and subsequently adjusted $P_{\mathrm{ETC}O_{2}}$ at 1400–5160 m for each participant measurement during ascent (see Figure 3).

Morning urine pH measurements were obtained on a subset of participants in both NAz (*n* = 35) and Az (*n* = 18) groups with ascent on some measurement days (1400, 3440, 3820, 4240, and either 4910 m (Az group) or 5160 m (NAz group)). Participants provided a sample of their first morning urination into a new, clean 110 mL sample container with a screw cap that could be secured immediately following collection and analysed within 5–30 min. Urine pH was measured aerobically using a pH meter and biological probe (B10P; VWR; sympHony, Edmonton, Canada), calibrated daily using standard pH buffers (3 and 7), and automatically temperature corrected. These measures are utilized here *post hoc* to confirm acetazolamide status (e.g., Cates et al., 2022; Galdston, 1955).

#### 2.3.4 Acetazolamide use

Following a characterization of VA with ascent in the complete group in Part A, we subsequently compared the NAz group with a separate group taking a prophylactic dose of oral acetazolamide (125 mg BID) following the same ascent profile (Part B). All expedition participants independently obtained a supply of acetazolamide via a prescription from their personal physician prior to the departure for the expeditions. For the expeditions where participants were not taking oral prophylactic acetazolamide, it was available for treatment of AMS symptoms under the guidance of the organization team. Participants included in the NAz group did not take prophylactic acetazolamide nor corticosteroids for treatment of AMS at any time during the incremental ascent to 5160 m. For the expedition where participants were taking acetazolamide (Az), participants self‐administered an oral prophylactic dose (125 mg BID) during ascent, as per accepted guidelines (e.g., Basnyat et al., 2003; Luks et al., 2019; van Patot et al., 2008). The Az group began taking acetazolamide on day 1 of ascent from 1400 to 2840 m (flight to Lukla airport and first trekking day) and continued self‐administration twice daily (morning and night) as per instructions from the expedition organizers during ascent.

For transparency, this observational retrospective evaluation between NAz and Az groups was not planned a priori. Rather, the unique opportunity to assess the potential effects of a self‐administered oral prophylactic dose of acetazolamide arose *post hoc*, given that data collection occurred across several HA research expeditions, where one group was taking oral acetazolamide as a part of the safety precautions of the expedition, and another group was not. Given that (a) this was not a planned drug intervention study (i.e., clinical trial), (b) the acetazolamide was obtained by each participant individually in advance via prescription from their own personal physicians, and (c) the drug use took place outside of Canada (Nepal), Health Canada approval was not required, as it is outside the scope of Part C, Division 5 of the Food and Drug Regulations. Thus, a Clinical Trial Application was not required to be submitted for review in advance (Health Canada, personal communication).

#### 2.3.5 Statistical analysis

For Part A, to assess the effects of altitude on $P_{\mathrm{ETC}O_{2}}$ and $S_{pO_{2}}$ for all the participants (*n* = 46) and groups with varying degrees of acclimatization, one‐factor repeated‐measures ANOVA was performed. Where significant *F*‐ratios were detected, a Tukey's *post hoc* test was performed for pair‐wise comparisons.

Fenn diagrams were constructed for each individual participant using daily $P_{\mathrm{ETC}O_{2}}$ measurements plotted against simultaneously measured $S_{pO_{2}}$. Given these responses were not linear, AUC values were calculated using the trapezoid method via the AUC function in GraphPad 9 Prism (GraphPad Software, Boston, MA, USA). To differentiate AUC between all participants in the NAz group (Part A), the magnitudes were ordered from smallest to largest, and divided into tertiles: large, medium and small demonstrating least (*n* = 15), moderately (*n* = 16) and highly acclimatized participants (*n* = 15), respectively. To subsequently compare AUC values between males (*n* = 25) and females (*n* = 21), an unpaired two‐tailed Student's *t*‐test was used.

To determine the changes in AMS scores during HA ascent, a non‐parametric, repeated‐measures Friedman's test was performed for all the participants, and groups with varying degrees of acclimatization. *Post‐hoc* Dunn's multiple comparison test was also performed to assess differences in AMS scores between different altitudes. To assess the differences in worst (i.e., highest) reported AMS scores between participants with low, moderate and high degree of acclimatization, a non‐parametric Kruskal–Wallis ANOVA was performed. The same procedure was repeated for total AMS scores after summing daily scores throughout the ascent (i.e., cumulative AMS score for each participant; Holmström et al., 2019).

For Part B, AUC was compared between the participants in Part A (*n* = 46; NAz) and the participants recruited in Part B, who were taking a prophylactic oral dose of acetazolamide (Az; *n* = 20). Modified Fenn diagrams were constructed in an identical fashion to Part A, and NAz and Az groups were compared using an unpaired two‐tailed *t*‐test, with effect size being calculated using Hedges’ *g* (given the defences in sample size).

AMS scores of the Az group were analysed similarly to the NAz participants, whereby non‐parametric, repeated‐measures Friedman's test was performed with *post hoc* Dunn's multiple comparison test to assess differences in AMS scores between different altitudes. The same procedure was repeated for total AMS scores after summing daily scores throughout the ascent (i.e., cumulative AMS score for each participant with ascent; Holmstrom et al., 2019).

To confirm acetazolamide status in Az group participants, urine pH was compared between NAz and Az groups with ascent using a mixed two‐factor ANOVA, which was repeated‐measures for altitude and non‐repeated‐measures for drug status, and a Tukey's *post hoc* test was performed for pairwise comparison where significant *F*‐ratios were detected.

In the group of NAz participants at 5160 m, two participants who took a single oral treatment dose of acetazolamide (250 mg) due to AMS symptoms were subsequently confirmed to be statistical outliers, as they fell outside the interquartile rage of a group of 37 participants with urine pH measures at that altitude, illustrating the effect of a treatment dose of Az on urine pH.

All data plots and statistical analysis were performed on GraphPad 9 Prism software.

## 3 RESULTS

### 3.1 Part A. Characterizing AUC on modified Fenn diagrams to assess VA with ascent

#### 3.1.1 Participant demographics

For Part A, we included data from 25 male participants and 21 female participants for a total of 46 participants, with a mean age of 27.7 ± 10.1 years and a mean body mass index (BMI) of 24.2 ± 3.6 kg/m^2^.

#### 3.1.2 $S_{pO_{2}}$ and $P_{\mathrm{ETC}O_{2}}$ changes with ascent in all participants

**FIGURE 4:**
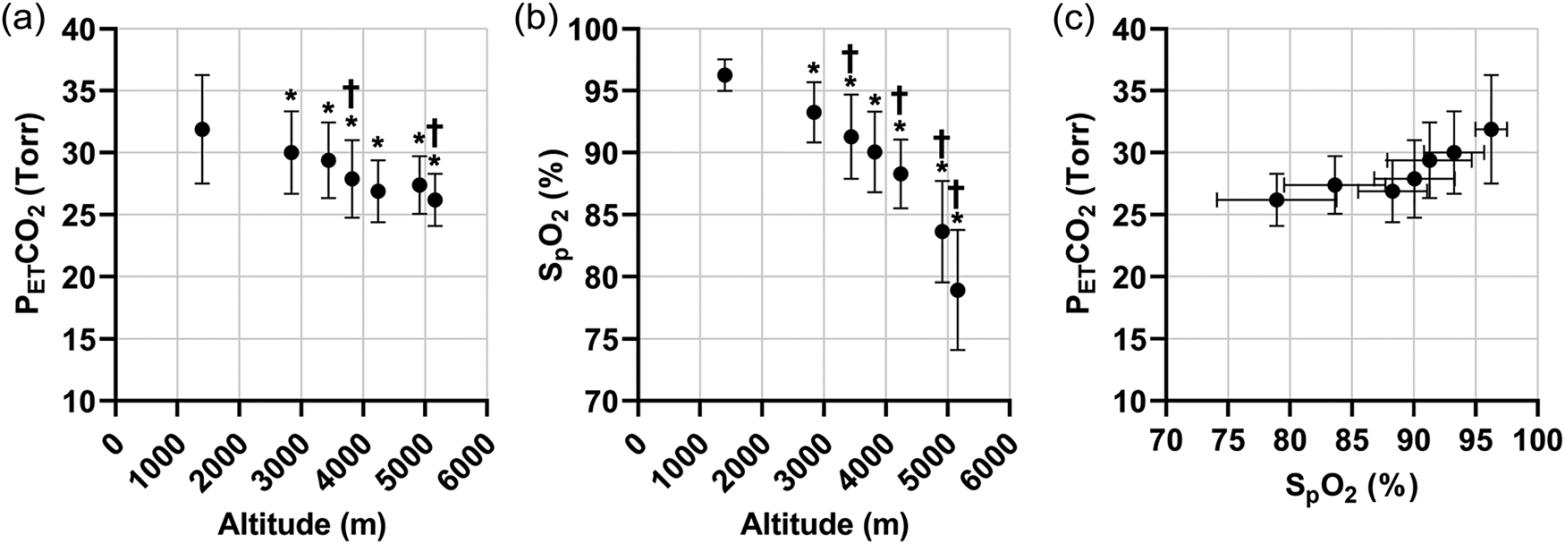
Changes in end‐tidal partial pressure of CO_2_, peripheral oxygen saturation and modified Fenn diagrams for all participants with incremental ascent. (a) End‐tidal partial pressure of CO_2_ ($P_{\mathrm{ETC}O_{2}}$; Torr) with incremental ascent. (b) Peripheral oxygen saturation ($S_{pO_{2}}$; %) with incremental ascent. (c) Modified Fenn diagram for all participants using $P_{\mathrm{ETC}O_{2}}$ and $S_{pO_{2}}$ with incremental ascent from (a) and (b). Data presented as means ± SD (*n* = 46). *Significantly different from measurements attained at 1400 m, *P *< 0.05. †Significantly different from the preceding attitude, *P *< 0.05.

For all participants in the NAz group (*n* = 46), mean $P_{\mathrm{ETC}O_{2}}$ values significantly decreased (*P *< 0.0001) with ascent, consistent with an HVR and further VA, as expected (see Figure 4a). Similarly, there were significant decreases (*P *< 0.0001) in mean $S_{pO_{2}}$ values due to the reduced oxygen availability at higher altitudes, as expected (see Figure 4b). Corresponding measurements obtained for mean $P_{\mathrm{ETC}O_{2}}$ and $S_{pO_{2}}$ were then plotted on a modified Fenn diagram in Figure 4c, which is qualitatively similar (i.e., following a similar pattern) in responses to the data published by Rahn and Otis (1949).

#### 3.1.3 Quantifying degree of ventilatory acclimatization

**FIGURE 5:**
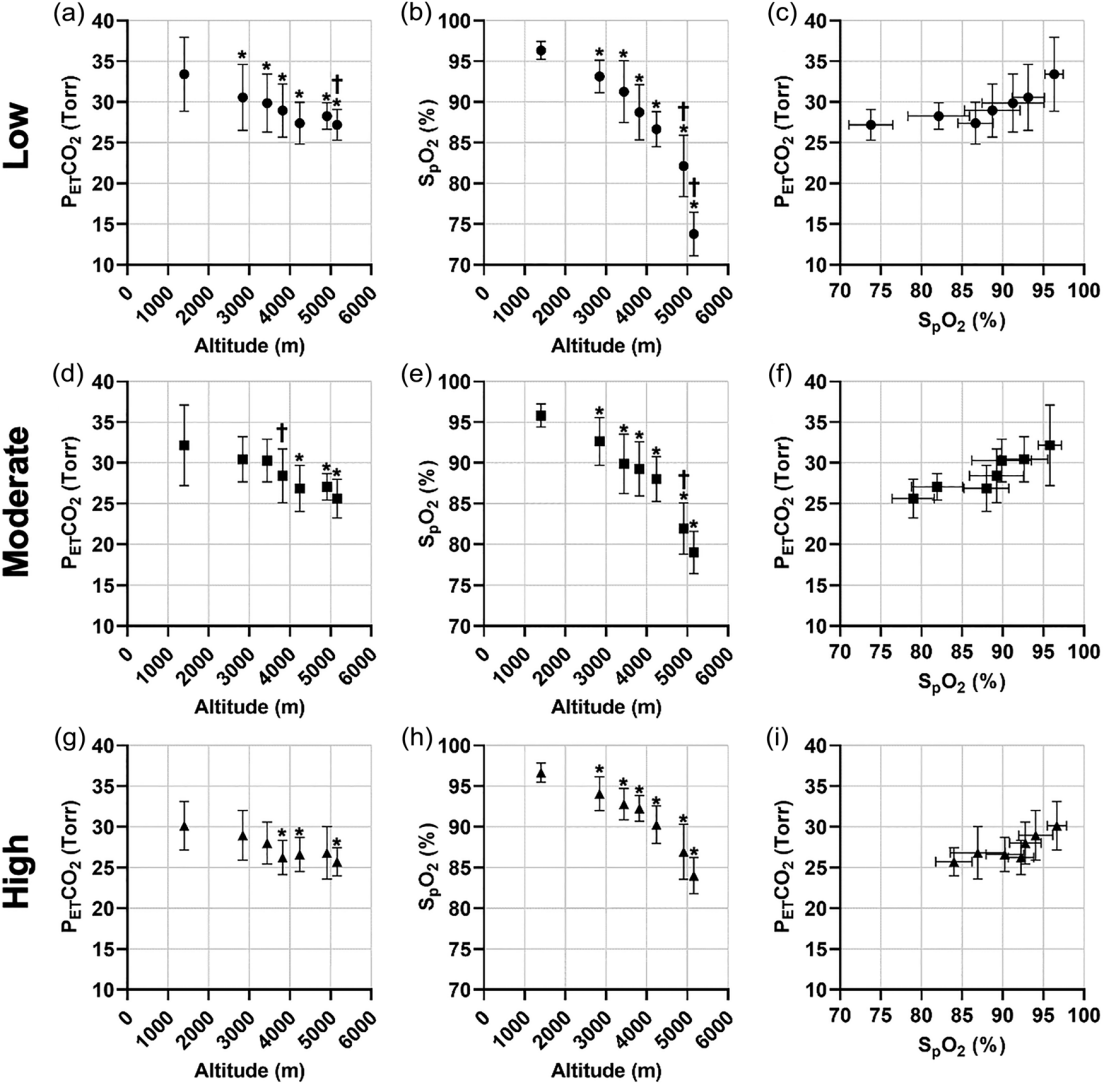
Changes in end‐tidal partial pressure of CO_2_, peripheral oxygen saturation and modified Fenn diagrams for participants with low, moderate and high degree of acclimatization. End‐tidal partial pressure of CO_2_ ($P_{\mathrm{ETC}O_{2}}$; Torr) and peripheral oxygen saturation ($S_{pO_{2}}$; %) and modified Fenn diagrams plotted against changes in altitude (m) during HA ascent for participants with low (a, d, g; *n* = 15), moderate (b, e, h; *n* = 16) and high (c, f, i; *n* = 15) degree of acclimatization (using trichotomized splits from Fenn diagram AUC). Data shown as means ± SD. *Significantly different from measurements attained at 1400 m, *P *< 0.05. †Significantly different from the preceding attitude, *P *< 0.05.

After performing a trichotomized split based on the magnitude of AUC and grouping participants into low, moderate and high degree of acclimatization cohorts, changes in $P_{\mathrm{ETC}O_{2}}$ and $S_{pO_{2}}$ were subsequently assessed. For all three groups, there were significant reductions (*P *< 0.0001) in $P_{\mathrm{ETC}O_{2}}$ measurements with ascent, as shown by Figure 5a, d, g. A similar trend was observed for $S_{pO_{2}}$, whereby significant reductions (*P *< 0.0001) were noted with increasing altitudes for the three cohorts (see Figure 5b, e, h). Corresponding measurements obtained for mean $P_{\mathrm{ETC}O_{2}}$ and $S_{pO_{2}}$ were then plotted on modified Fenn diagrams for each group in Figure 5c, f, i.

#### 3.1.4 Area under the curve of modified Fenn diagrams

To calculate the AUC for the least, moderately and highly acclimatized participants, modified Fenn diagrams were constructed for the three groups using $P_{\mathrm{ETC}O_{2}}$ and $S_{pO_{2}}$ measurements with ascent. As illustrated in Figure 5c, f, i the least acclimatized cohort had an upward and leftward shift on the Fenn diagram, compared to the moderate group. This trend is further pronounced in the highly acclimatized individuals, with $P_{\mathrm{ETC}O_{2}}$ and $S_{pO_{2}}$ values shifted downwards and rightwards on the diagram (see Figure 5i).

**FIGURE 6:**
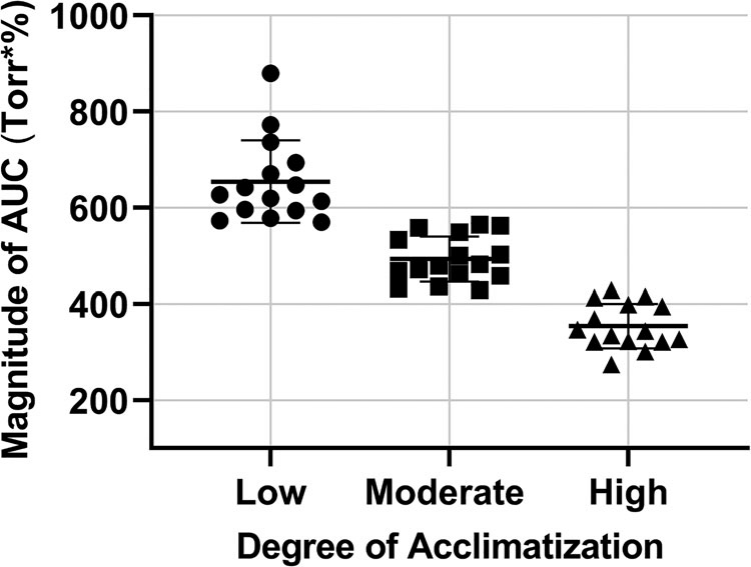
Quantification and comparison of magnitudes of the areas under the curve (AUC) in Torr·% obtained from individually constructed Fenn diagrams for participants with low, moderate and high degree of acclimatization. Magnitudes of AUC (Torr·%) acquired from individually constructed Fenn diagrams using peripheral oxygen saturations ($S_{pO_{2}}$; %) and end‐tidal partial pressures of CO_2_ ($P_{\mathrm{ETC}O_{2}}$; Torr; see Figure 4). Values reported as means ± SD with circles representing individual participants with low degree of acclimatization, and squares and triangles represent moderately and highly acclimatized participants, respectively.

The mean AUC values during ascent to 5160 m for participants with low, moderate and high degree of acclimatization were 654.2 ± 86.1, 493.0 ± 47.2 and 354.3 ± 46.4 Torr·%, respectively, demonstrating that the more acclimatized individuals were, the smaller their AUC was on the modified Fenn diagram (see Figure 6), illustrating values for a range of acclimatization using this novel analysis. Here, we provide additional values for VA using AUC on modified Fenn diagrams using published data from other expeditions that performed an incremental ascent to HA (see Table 2).

**TABLE 2:** Comparison of normative VA values obtained from AUC calculations on modified Fenn diagrams from previous studies employing incremental ascent.

Study	Altitude (m)	Ascent days	Number of participants	Mean AUC (Torr^a^%)	Measurements used
Grant et al. (2002)					
Acute exposure^a^	4850	—	22	611.8	$S_{aO_{2}}$ and $P_{\mathrm{ETC}O_{2}}$
Expedition	4320	17	22	407.9	$S_{aO_{2}}$ and $P_{\mathrm{ETC}O_{2}}$
Willie et al. (2014)^b^	5050	6–8	7–13	430.5	$S_{pO_{2}}$ and $P_{\mathrm{ETC}O_{2}}$
Burgess et al. (2004)^b^	5050	12	13–14	504.7	$S_{cO_{2}}$ and $P_{\mathrm{aC}O_{2}}$
Willie et al. (2018)					
Lowlanders	5050	9–10	21	570.1	$S_{aO_{2}}$ and $P_{\mathrm{aC}O_{2}}$
Sherpa	5050	9–10	11	609.0	$S_{aO_{2}}$ and $P_{\mathrm{aC}O_{2}}$
Current study—Part A					
All	5160	10	46	500.4	$S_{pO_{2}}$ and $P_{\mathrm{ETC}O_{2}}$
Low	5160	10	15	654.2	$S_{pO_{2}}$ and $P_{\mathrm{ETC}O_{2}}$
Moderate	5160	10	16	493.0	$S_{pO_{2}}$ and $P_{\mathrm{ETC}O_{2}}$
High	5160	10	15	354.3	$S_{pO_{2}}$ and $P_{\mathrm{ETC}O_{2}}$
Current study—Part B^b^	5160	10	20	384.0	$S_{pO_{2}}$ and $P_{\mathrm{ETC}O_{2}}$
Imray et al. (2005)	5260	9	9	478.9	$S_{pO_{2}}$ and $P_{\mathrm{ETC}O_{2}}$
Rahn and Otis (1949)					
Acute exposure^a^	6706	—	22	1251.0	$S_{cO_{2}}$ and $P_{\mathrm{aC}O_{2}}$
Acclimatized^c^	6949	Variable	Variable	697.9	$S_{cO_{2}}$ and $P_{\mathrm{aC}O_{2}}$
Sutton et al. (1988)^a^	8848	40	8	829.3	$S_{aO_{2}}$ and $P_{\mathrm{aC}O_{2}}$

Data shown for this and other incremental ascent studies, listed from lowest to highest maximum altitudes. Our study includes values for all the participants (*n* = 46), which were subsequently divided into groups of low (*n* = 15), moderate (*n *= 16) and high (*n* = 15) degree of acclimatization. Other studies are subdivided based on different conditions or groups. In cases where $S_{pO_{2}}$ or $S_{aO_{2}}$ measurements were not available, the Severinghaus transform (Severinghaus, 1979) was applied to calculate oxygen saturation ($S_{cO_{2}}$) from $P_{aO_{2}}$. Similarly, if $P_{\mathrm{ETC}O_{2}}$ data were not reported, $P_{\mathrm{aC}O_{2}}$ was used instead. ^a^Data obtained from simulated hypoxic conditions in a laboratory. ^b^Prophylactic oral acetazolamide use during ascent up to 5160 m. ^c^Data obtained from the line‐of‐best‐fit, as multiple studies were used.

#### 3.1.5 Comparison between sexes

For all the participants, there were no significant differences (*P* = 0.94) in AUC between male (*n* = 25) and female (*n* = 21) trekkers, which had mean values of 501.7 ± 140.3 and 498.7 ± 136.1 Torr·%, respectively.

#### 3.1.6 Acute mountain sickness scores

Non‐parametric measures that determine the median and the interquartile range (IQR; 25th–75th percentile) were used to compare qualitative AMS scores. No statistically significant differences (*P* = 0.75) were found in the worst reported AMS scores between the least, moderately and highly acclimatized participants. Similarly, there were no statistically significant differences (*P* = 0.84) in the cumulative AMS scores between participants with low, moderate and high degree of acclimatization.

#### 3.1.7 Urine pH

Urine pH was unchanged with ascent from baseline (i.e., 1400 m) values (see Table 3), suggesting that aerobic urine pH measures lack utility in assessing acid–base status during acclimatization in acetazolamide‐free individuals.

**TABLE 3:** Mean changes in ancillary variables during HA ascent over 10 days between NAz and Az groups.

Variable	1400 m Day 0	2840 m Day 2	3440 m Day 3	3820 m Day 5	4240 m Day 7	4910 m Day 9	5160 m Day 10	*P*
*P*_ATM_ (mmHg)	642	537	497	473	448	410	397	NA
$P_{IO_{2}}$ (mmHg)	125	103	95	90	84	76	74	NA
$S_{pO_{2}}$ (%; mean ± SD)								
NAz (*n* = 46)	96.26 ± 1.27	93.26 ± 2.42^a^	91.28 ± 3.41^a,b^	90.07 ± 3.25^a^	88.30 ± 2.80^a,b^	83.63 ± 4.08^a,b^	78.93 ± 4.83^a,b^	<0.0001
Az (*n* = 20)	95.80 ± 1.28	94.35 ± 1.63	92.85 ± 2.60^a^	90.60 ± 2.78^a^	90.20 ± 2.76^a^	84.05 ± 3.97^a,b^	81.10 ± 5.05^a,b^	<0.0001
$P_{\mathrm{ETC}O_{2}}$ (Torr; mean ± SD)								
NAz (*n* = 46)	31.91 ± 4.38	30.00 ± 3.34^a^	29.40 ± 3.07^a^	27.88 ± 3.13^a,b^	26.95 ± 2.50^a^	27.37 ± 2.33^a^	26.15 ± 2.10^a,b^	<0.0001
Az (*n* = 20)	30.61 ± 3.05	25.37 ± 2.21^a^	24.82 ± 2.3^a^	25.14 ± 2.15^a^	24.62 ± 1.93^a^	24.47 ± 2.21^a^	25.55 ± 1.5^a^	<0.0001
Daily AMS score [median (range)]								
NAz (*n* = 46)	0(0–1)	0(0–3)	0(0–3)	0(0–3)	0(0–3)	1(0–3)^a^	1(0–6)^a^	<0.0001
Az (*n* = 20)	0(0–1)	0(0–2)	0(0–2)	0(0–2)	0(0–2)	1(0–4)	1(0–6)^a^	<0.0001
Aerobic urine pH (mean ± SD)								
NAz (*n* = 35)	6.05 ± 0.55	N/A	6.04 ± 0.52	5.88 ± 0.50	6.00 ± 0.39	5.90 ± 0.47		0.29
Az (*n* = 18)	5.94 ± 0.55	N/A	6.65 ± 0.53^a^	6.63 ± 0.56^a^	6.74 ± 0.42^a^	6.74 ± 0.53^a^		<0.0001

Data shown for all the participants in the NAz group (*n* = 46) and Az group (*n* = 20). Aerobic urine pH measures were obtained on subset of participants with ascent at most locations. Due to logistical constraints, note that the NAZ group was measured at 5160 on day 10, whereas the Az group was measured at 4910 m on day 9, and are treated together as ∼5000 m. ^a^Significantly different from measurements attained at 1400 m, *P *< 0.05. ^b^Significantly different from the preceding altitude, *P *< 0.05. Abbreviations: AMS, acute mountain sickness; *P*_ATM_, atmospheric pressure; $P_{\mathrm{ETC}O_{2}}$, partial pressure of end‐tidal carbon dioxide; $P_{IO_{2}}$, partial pressure of inspired oxygen; $S_{pO_{2}}$, peripheral oxygen saturation. NA, Not applicable.

### 3.2 Part B. Comparing AUC on modified Fenn diagrams between NAz and Az groups

#### 3.2.1 Participant demographics

For Part B, we included data from 7 males and 13 females for a total of 20 participants, with an average age of 27.7 ± 8.3 years and a mean BMI of 25.1 ± 4.7 kg/m^2^.

#### 3.2.2 $S_{pO_{2}}$ and $P_{\mathrm{ETC}O_{2}}$ changes with ascent

**FIGURE 7:**
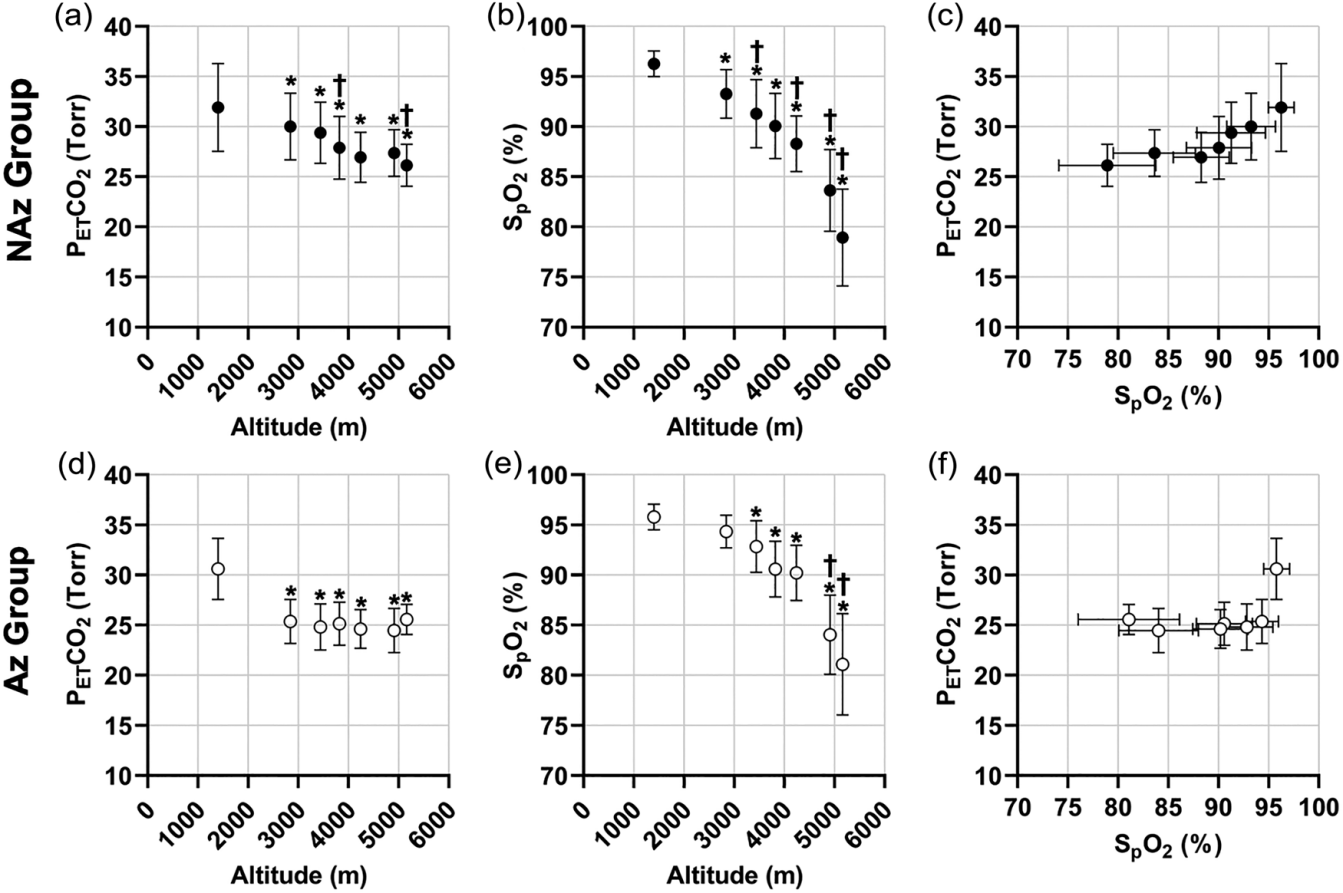
Changes in end‐tidal partial pressure of CO_2_, peripheral oxygen saturation and modified Fenn diagrams for NAz and Az groups with incremental ascent. (a, d) End‐tidal partial pressure of CO_2_ ($P_{\mathrm{ETC}O_{2}}$; Torr) with incremental ascent. (b, e) Peripheral oxygen saturation ($S_{pO_{2}}$; %) with incremental ascent. (c, f) Modified Fenn diagram for all participants using $P_{\mathrm{ETC}O_{2}}$ and $S_{pO_{2}}$ with incremental ascent from (a) and (b). Data presented as means ± SD. Filled circles represent NAz group (*n* = 46), and open circles represent Az group (*n* = 20). *Significantly different from measurements attained at 1400 m, *P *< 0.05. †Significantly different from the preceding attitude, *P *< 0.05.

For comparison, the NAz group $P_{\mathrm{ETC}O_{2}}$, $S_{pO_{2}}$ and Fenn diagram are illustrated in 7a‐c (also see 4a‐c). Similarly, there were significant decreases in both mean $P_{\mathrm{ETC}O_{2}}$ (*P *< 0.0001; see Figure 7d) and mean $S_{pO_{2}}$ (*P *< 0.0001; see Figure 7e) measurements in the Az group as it ascended to HA. These values were then plotted on a modified Fenn diagram in Figure 7f and compared against the NAz group. Although both groups followed a decreasing trend in mean $P_{\mathrm{ETC}O_{2}}$ and $S_{pO_{2}}$ values, participants using acetazolamide reached lower $P_{\mathrm{ETC}O_{2}}$ more quickly (due to increases in ventilation), allowing them to maintain relatively higher $S_{pO_{2}}$ values, resulting in a more rightward and downward trend on the modified Fenn diagram (see Figure 7f).

#### 3.2.3 Quantifying the degree of ventilatory acclimatization

**FIGURE 8:**
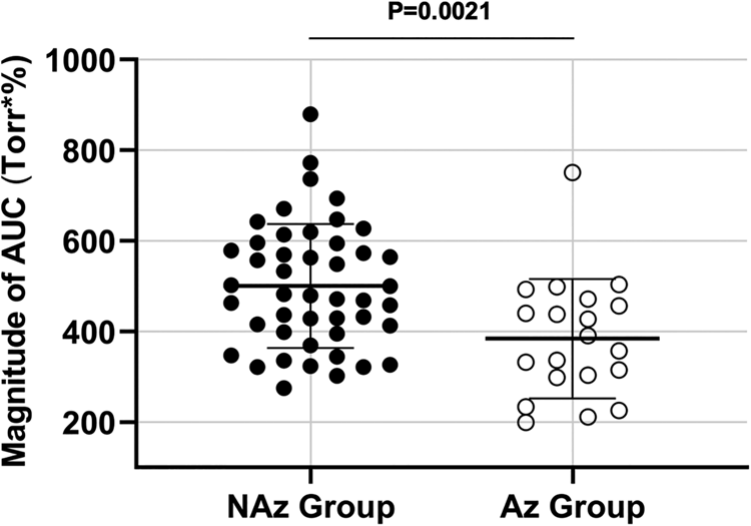
Quantification and comparison of magnitudes of the areas under the curve (AUC) in Torr·% obtained from individually constructed Fenn diagrams for participants in the NAz and Az groups. Magnitudes of AUC (Torr·%) acquired from individually constructed Fenn diagrams using peripheral oxygen saturations ($S_{pO_{2}}$; %) and end‐tidal partial pressures of CO_2_ ($P_{\mathrm{ETC}O_{2}}$; Torr; see Figure 6). Values reported as means ± SD with filled circles representing individual participants in the NAz group (*n* = 46) and open circles represent individual participants in the Az group (*n* = 20). Actual *P*‐value reported on graph.

To compare the mean AUC values between NAz and Az groups, average $P_{\mathrm{ETC}O_{2}}$ and $S_{pO_{2}}$ measurements were used to construct modified Fenn diagrams from which the AUCs were calculated and averaged. There was a significant difference (*P* = 0.0021; Hedges’ *g* = 0.86) between the mean AUC values of both groups. As demonstrated by Figure 8, the Az participants had a smaller average AUC value (384.0 ± 132.0 Torr·%) when compared to the average NAz value (500.3 ± 136.9 Torr·%), demonstrating that participants taking oral Az generally have a smaller AUC on modified Fenn diagrams.

#### 3.2.4 Comparison between sexes

For the Az participants, there were no significant differences (*P* = 0.57) in AUC between male (*n* = 7) and female (*n* = 13) trekkers, with mean values of 407.5 ± 76.7 and 371.3 ± 155.4 Torr·%, respectively.

#### 3.2.5 Acute mountain sickness scores

Non‐parametric measures that determine the median and the IQR were used to compare qualitative AMS scores between the NAz and the Az groups. No statistically significant differences (*P* = 0.2) were found in the worst reported AMS scores between the two groups. Similarly, there were no statistically significant differences (*P* = 0.7) in the cumulative AMS scores between the participants who used acetazolamide versus those who did not.

#### 3.2.6 Urine pH

**FIGURE 9:**
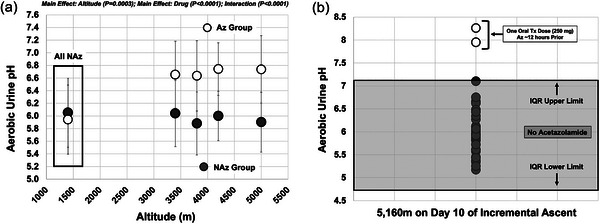
Aerobic urine pH with ascent to assess and confirm acetazolamide (Az) status. (a) Participant aerobic urine pH data were grouped into altitude categories of 1400 m (day 0; baseline; all Az‐free), 3400 m (day 3), 3800 m (day 5), 4200 m (day 7), 5000 m (day 9/10) in both NAz (filled circles; *n* = 35) and Az (open circles; *n* = 18) for comparison. Main and interaction effects and associated *P*‐values noted on graph. (b) Urine pH measurements on morning 10 of ascent at 5160 m between NAz (filled circles; *n* = 35) and following a single treatment dose of Az (open circles; *n* = 2). Two participants took a treatment dose of oral Diamox (250 mg) for acute mountain sickness symptoms at approximately 19.00 h after arrival at 5160 m, approximately 12 h before urine pH measures. The grey box represents the lower and upper interquartile ranges (IQR) for urine pH in the entire group of *n* = 37. The two Az participants are outside the upper IQR limit (i.e., statistical outlier).

Urine pH was immediately alkalinized with use of oral acetazolamide following baseline values with superimposed ascent (Table 3), but was unchanged with further ascent, similar to the NAz group. However, urine pH in the Az group was more alkaline than that in the NAz group throughout the ascent (main effect altitude, *P* = 0.0003; main effect drug, *P *< 0.0001; interaction, *P *< 0.0001; see Figure 9a). Similarly, two participants who took a single oral treatment dose of acetazolamide (250 mg) the night prior had significantly more alkaline urine pH than the rest of the group ∼07.00 h the next morning, as they were statistical outliers, falling outside the IQR for the group (Figure 9b). These data suggest that aerobic urine pH can differentiate acetazolamide status in healthy participants (also see: Cates et al., 2022; Galdston, 1955).

## 4 DISCUSSION

We aimed to characterize VA using portable devices and modified Fenn diagrams by plotting $P_{\mathrm{ETC}O_{2}}$ against $S_{pO_{2}}$ during incremental HA ascent to 5160 m in the Nepal Himalayas in a large trekking group. First, we characterized a range of normative values for AUC derived from modified Fenn diagrams for low, moderately and highly acclimatized individuals, and compared these values to those calculated from published studies using similar metrics with incremental ascent. Second, we characterized AUC in a group taking a prophylactic dose of acetazolamide throughout an identical ascent profile, and demonstrated a smaller AUC than an acetazolamide‐free group, similar to the highly acclimatized cohort in our initial characterization group.

### 4.1 Modified Fenn diagrams to assess ventilatory acclimatization

Rahn and Otis (1949) demonstrated that previously acclimatized participants had relatively higher $P_{AO_{2}}$ and lower $P_{\mathrm{AC}O_{2}}$ during progressive hypoxia because of VA, compared to unacclimatized participants during acute hypoxia. Because of this response, and higher ventilatory sensitivity to decreases in $P_{aO_{2}}$, the acclimatized cohort had larger HVR magnitudes as oxygen levels were gradually decreased, resulting in a downward and rightward shift on the Fenn diagram (Rahn & Otis, 1949). However, Rahn and Otis did not provide normative quantitative VA values for individuals with varying degrees of acclimatization, and a method to quantitatively compare the degree of acclimatization between individuals was not advanced. Therefore, we quantified VA during incremental ascent to 5160 m using portable devices to measure $P_{\mathrm{ETC}O_{2}}$ and $S_{pO_{2}}$, and constructed modified Fenn diagrams to assess VA magnitude using AUC measures, potentially bypassing the methodological limitations of previous experiments assessing the HVR where complex laboratory equipment is required (e.g., Steinback & Poulin, 2007).

Our study is the first to provide values for VA by calculating AUC on modified Fenn diagrams, which relies only on portable $P_{\mathrm{ETC}O_{2}}$ and $S_{pO_{2}}$ measurements. We demonstrate that highly acclimatized participants have a smaller AUC during incremental ascent, and that modified Fenn diagrams may be effectively used to assess VA in large trekking or climbing cohorts, both with and without Az. As expected, our data demonstrate that for all participants, both $S_{pO_{2}}$ and $P_{\mathrm{ETC}O_{2}}$ decreased during the ascent, the latter due to increased ventilation in response to reduced oxygen availability (i.e., HVR). Our data illustrate that decreases in $S_{pO_{2}}$ were more apparent in participants with low and moderate degrees of VA, compared to highly acclimatized individuals. Despite smaller differences in $P_{\mathrm{ETC}O_{2}}$, $S_{pO_{2}}$ variability caused significant differences in AUC between the three groups. Other studies also noted decreased $P_{\mathrm{ETC}O_{2}}$ and improved $S_{pO_{2}}$ in more acclimatized participants due to greater VA and increased ventilation (Katayama et al., 2005; Kong et al., 2015; Tannheimer et al., 2013), confirming that our results are physiologically congruent, and that highly acclimatized participants have smaller AUCs on modified Fenn diagrams (Rahn & Otis, 1949).

Lastly, unlike current laboratory HVR tests, which expose participants to transient hypoxia and require complex equipment that may lack feasibility in fieldwork contexts, our method uses portable devices, which in many cases are more practical. This method is well suited for use in large trekking or climbing groups during HA ascent to assess VA given its portability and simplicity, and availability of normative values for comparison (Table 2). Consequently, we suggest that AUC on modified Fenn diagrams could be applied during HA trekking, climbing and research expeditions to assess VA.

### 4.2 Sex differences in HVR and ventilatory acclimatization

There is conflicting literature on the influence of biological sex on the acute HVR and VA (Bhaumik et al., 2003; Camacho‐Cardenosa et al., 2022; Chapman et al., 2010; Goldberg et al., 2017). For instance, Bhaumik et al. (2003) found no significant sex differences in HVR during and after an ascent to 4300 m, while Camacho‐Cardenosa et al. (2022) reported a more pronounced HVR and improved $S_{pO_{2}}$ in males compared to females during a 7 h exposure to normobaric hypoxia. Nevertheless, our study did not find any significant differences in AUC between male and female participants in either NAz or Az groups, suggesting that there was no difference in VA during incremental ascent based on biological sex in our cohorts.

### 4.3 AMS scores

Although highly acclimatized participants had smaller AUCs on modified Fenn diagrams, our study did not find a relationship between AUC magnitudes and AMS scores. There were no significant differences for worst reported and cumulative AMS scores using the Lake Louise Questionnaire (Roach et al., 2018) between the participants with low, moderate and high degree of acclimatization, nor were there differences between NAz and Az groups. It is likely that a potential relationship was not apparent, in part, because of the subjective nature of AMS assessments (e.g., Wagner et al., 2012). Roach and Kayser (2007) suggest that it is particularly difficult to interpret AMS due to a lack of clinical signs, and therefore, one must rely solely on subjective symptom rating by the participant. The authors also state that AMS scoring is inadequate for between‐subject comparisons, as these are highly subjective and differ from person to person, thus compromising the statistical analysis of these non‐parametric data. Furthermore, Frühauf et al. (2016) state that some symptoms, such as headache and fatigue, may be caused by other factors that are not related to HA (e.g., exercise, dehydration). Although some studies report relationships between physiological variables and AMS scores (e.g., Leacy et al., 2021), quantitative analysis of the relationships of physiological measures and self‐reported AMS remains controversial, and is of likely limited utility.

The magnitude of AUC was not related to the severity AMS in our study, possibly because participants performed a slow, incremental ascent, which allowed the participants to acclimatize to progressively lower oxygen levels with ascent. It is well known that faster ascent rates are likely to result in more severe AMS symptoms (Roach et al., 2018; Wang et al., 2010). During the ascent, participants had limited symptom manifestation and severity, reflected in lower reported scores (see Tables 1 and 3). The updated Lake Louise Questionnaire considers presence of AMS only if the score is greater than three and a headache is present (Roach et al., 2018). However, because most of the reported scores were low and increased only towards the end of the ascent (i.e., ∼5000 m), we also assessed a novel technique of quantifying AMS, which included summing all the scores throughout the ascent in addition to assessing the highest reported score (Holmstrom et al., 2019), to further differentiate participants. Although we do not know the effectiveness nor the validity of this method, it may be used for incremental ascent profiles where individual daily AMS scores are low or zero. Additional research is required to determine the validity and effectiveness of this cumulative score method, and the potential relationship between VA and AMS symptom manifestation.

### 4.4 Comparison with previous studies

Using data from other expeditions that performed simulated or incremental ascent models, we constructed modified Fenn diagrams and calculated AUC values, providing additional values for different ascent durations and maximum altitudes (see Table 2). In these studies, as the maximum altitude increased, so did the mean AUC values. This was likely in part because of decreased oxygen saturation with ascent, which pushed the $S_{pO_{2}}$ values to the left, and ultimately increased the AUC on modified Fenn diagrams (e.g., Sutton et al., 1988). Trekking, climbing or longer exposure duration also increased VA compared to laboratory studies, because participants had more time to acclimatize to HA during longer ascent profiles. This additional exposure may potentially explain the striking differences in AUC between acute simulated and hypobaric hypoxic conditions in the same individuals, whereby actual ascent reduced the AUC (e.g., Grant et al., 2002). Based on the values obtained from Rahn and Otis (1949), it is evident that previously acclimatized participants have smaller AUCs, compared to unacclimatized participants, which were exposed to acute hypoxia only in the hypobaric chambers. Interestingly, Willie et al. (2018) compared lowlanders and Sherpa using a similar ascent profile to our study. Not only were the groups’ mean AUC values similar in magnitude to our study, but they were similar to each other, consistent with the suggestion that Sherpa have similar HVR and/or VA to lowlanders with ascent (Gilbert‐Kawai et al., 2014). Ultimately, the potential utility of our method lies in comparing individuals or groups of individuals engaging in a similar incremental ascent profile to assess differential VA across the group.

### 4.5 Effect of acetazolamide

Acetazolamide is commonly used as a prophylactic and/or a therapeutic drug to treat AMS by helping to improve arterial oxygen saturation by creating a state of metabolic acidosis, further increasing ventilation with ascent (Chakraborti et al., 1985; Swenson, 1998; Basnyat et al., 2006; Leaf & Goldfarb, 2007). Multiple studies (Basnyat et al., 2003; van Patot et al., 2008; Cates et al., 2022) have shown improved VA at HA with the use of acetazolamide, which was mainly characterized by decreased AMS scores, improved ventilation and greater oxygen saturation. Contrary to other studies, however (e.g., Basnyat et al., 2003; van Patot et al., 2008), we were not able to demonstrate a significant decrease in AMS scores with the use of acetazolamide, likely due to an incremental ascent design in our expeditions, which resulted in low AMS symptom scores. Although our study does not provide a direct comparison in ventilation between NAz and Az participants, augmented ventilation can be indirectly observed by decreased $P_{\mathrm{ETC}O_{2}}$ values in the Az group. Additionally, Az participants had a significantly smaller mean AUC value when compared to NAz group, suggesting greater VA. Our findings also demonstrate the validity of our metric as the AUC values of the Az group were most quantitatively similar to the highly acclimatized cohort in the NAz group, thus showing that smaller AUCs on modified Fenn diagrams indicate greater VA during incremental ascent.

### 4.6 Methodological considerations

Despite their common usage, current laboratory HVR assessments are impractical in many HA research expeditions. This is in part because they impose additional hypoxic stress on the participant in already low oxygen environments, and require sophisticated equipment that is difficult to transport to HA contexts (e.g., gas tanks, computers and gas analysers; e.g., Steinback & Poulin, 2007). Moreover, Pfoh et al. (2016; 2017) demonstrated that different laboratory assessments of the HVR did not elicit similar magnitude nor correlated responses, as transient tests elicit a smaller HVR compared to steady‐state hypoxia experiments, and the response magnitudes were not correlated within individuals. In addition, laboratory isocapnic HVR tests have little relationship to the steady‐state poikilocapnic ventilatory strategy employed when ascending to HA and breathing ambient air. Therefore, to mitigate these limitations, we used portable devices that allowed us to easily quantify VA using a modified Fenn diagram (using $S_{pO_{2}}$ instead of $P_{AO_{2}}$ or $P_{aO_{2}}$). Our method requires only a portable capnograph and finger pulse oximeter, thereby eliminating the need to transport cumbersome equipment and making HA fieldwork on large trekking groups more feasible. In addition, our method is considerably safer because it does not require the exposure of the participant to an additional hypoxic stimulus. Finally, our method does not attempt to isolate peripheral and central chemoreceptors from each other (e.g., Wilson & Teppema, 2016), allowing for a more integrative assessment of chemoreflex functioning in the steady‐state. Although these laboratory approaches may provide valuable information for the purposes of assessing specific mechanisms, our approach allows for greater ecological validity in the integrative assessment of overall steady‐state chemoreflex functioning in a high altitude expedition setting.

In our study, we corrected our $P_{\mathrm{ETC}O_{2}}$ measures against previously obtained $P_{\mathrm{aC}O_{2}}$ measures during an identical ascent profile (Zouboules et al., 2018). As we outline in the Methods, the portable capnograph we utilized for $P_{\mathrm{ETC}O_{2}}$ measures has a validated atmospheric pressure range up to ∼3200 m (see Methods). Thus, measures of $P_{\mathrm{ETC}O_{2}}$ above this altitude may be unreliable. Indeed, we showed that using this capnograph model with incremental ascent, the $P_{\mathrm{ETC}O_{2}}$ − $P_{\mathrm{aC}O_{2}}$ gradient is exaggerated with ascent to 5160 m (Zouboules et al., 2018). At sea level whilst breathing ambient air, $P_{\mathrm{ETC}O_{2}}$ is known to underestimate $P_{\mathrm{aC}O_{2}}$ by 1–2 Torr (e.g., Robbins et al., 1990). In addition, Ito et al. (2008) showed that across a range of oxygen tensions and respiratory rates, $P_{\mathrm{ETC}O_{2}}$ and $P_{\mathrm{aC}O_{2}}$ values were not statistically different from each other. Thus, with the assumption that the $P_{\mathrm{ETC}O_{2}}$ − $P_{\mathrm{aC}O_{2}}$ difference is unchanged while breathing ambient air at rest with ascent, we corrected for the exaggerated underestimation of our within‐individual $P_{\mathrm{ETC}O_{2}}$ data with ascent using a linear regression model from a large sample of $P_{\mathrm{aC}O_{2}}$ and $P_{\mathrm{ETC}O_{2}}$ values obtained during an identical ascent profile to 5160 m (Zouboules et al., 2018). However, plotting Fenn diagrams to calculate AUC and quantify ventilatory acclimatization can also be utilized using a variety of measures of CO_2_ and oxygenation, including arterial (e.g., Willie et al., 2018), capillary or hot hand venous (e.g., Krapf et al., 1991) or end‐tidal or alveolar (e.g., Grant et al., 2002; Imray et al., 2005; Rahn & Otis, 1949; Willie et al., 2014; see Table 2), depending upon the context, all of which can be portable on HA expeditions. This portability can further be extended to arterial blood measures with the use of an Abbott i‐STAT unit with batteries (see Bird, Leacy et al. 2021 and Zouboules et al., 2018).

Another limitation of our methods is that the normative values we provide for VA may differ considerably for expeditions utilizing a rapid ascent and residence model, where participants rapidly ascend and reside at a single HA over time (e.g., Bird, Leacy et al. 2021; Steele et al., 2022). In our study, analogous to the original demonstration of a Fenn diagram with acute hypoxic exposure (Rahn & Otis, 1949), we performed an incremental ascent model, whereby participants were exposed to incrementally higher hypoxic stress, but with acclimatization superimposed over the duration of the ascent profile. Therefore, quantification of AUC on modified Fenn diagrams to assess VA following rapid ascent to and residence at a single HA requires further investigation, and likely differs substantially from incremental ascent. Finally, our measurements were acquired during an ascent to 5160 m over 10 days, which might differ from expeditions that are longer in duration and/or that reach higher absolute altitudes, where oxygen availability is further decreased (e.g., Grocott et al., 2009), or in studies that utilize acetazolamide to aid in VA (e.g., current study; Tissot van Patot et al., 2008; Willie et al., 2014). Our comparison of published studies in Table 2, as well as Part B of the present study comparing NAz and Az groups with ascent, illustrates how these considerations may affect our novel analytical perspective. Lastly, our study and those reported in Table 2 are only from incremental ascent models, consistent with the original Fenn approach, with inspired oxygen being reduced incrementally, and ventilatory acclimatization taking place throughout. Indeed, some ascent profiles include a rapid ascent and residence at a single altitude for occupational or research purposes (e.g., Bird, Leacy et al. 2021), but it is unclear to what extent applying a Fenn approach to assessing ventilatory acclimatization in these ascent profiles may have utility, although this could be the focus of future investigations.

Regarding acid–base status, we acknowledge that blood acid–base status affects the relationship between $P_{O_{2}}$ and $S_{pO_{2}}$, given the known relationship between CO_2_/[H^+^] and oxyhemoglobin curve shifts, which may affect the use of $S_{pO_{2}}$ instead of $P_{\mathrm{ET}O_{2}}$. In a previous study following the identical ascent profile, participants were fully acclimatized from a renal compensation perspective up to ∼4300 m, but developed a mild respiratory alkalosis at ∼5200 m (Zouboules et al., 2018). There appears to be a threshold altitude, above which full renal compensation is no longer possible. That threshold appears to be ∼4500 m (e.g., Bird et al., 2021; Forster et al., 1975; Steele et al., 2022; Zouboules et al., 2018). Thus, as with all respiratory chemoreflex studies, measures of $P_{O_{2}}$ and/or $P_{CO_{2}}$ may not capture the whole picture when acid–base disturbances are superimposed. In addition, the acetazolamide group likely had a metabolic acidosis relative to the NAz group throughout ascent, but we do not have direct evidence of this aside from the urine pH data reported herein, which may have further affected the $S_{pO_{2}}$ values we utilized relative to $P_{aO_{2}}$. Caveats accepted, it remains the case that when comparing the two groups using our novel analytical method, our data are congruent with expected physiology, given that the Az group had a ∼23% lower AUC than the NAz group (*P* = 0.0021; Hedges' *g* = 0.86).

### 4.7 Assessing acetazolamide status using aerobic urine pH samples

Urine pH measures have been utilized previously to assess acid–base regulation (e.g., Ge et al., 2006; Gledhill et al., 1975; Galdston et al., 1955). However, in previous studies, urine samples were obtained anaerobically, which lacks feasibility in fieldwork contexts, and limits participant recruitment to males (e.g., Gledhill et al., 1975). Here, we sought to assess the feasibility of aerobic urine samples in a large participant pool of both males and females to assess acid–base acclimatization, and subsequently confirm acetazolamide status in those taking an oral prophylactic dose.

In a study conducted by Ge et al. (2006), urine pH was measured anaerobically at simulated moderate altitudes using a hypobaric chamber. The average baseline urine pH at low altitude (∼400 m) was 5.97, similar to our aerobically obtained measures at 1400 m. At 6 h at simulated 2800 m, the urine pH values rose to 6.69, suggesting bicarbonate excretion and/or systemic H^+^ retention in response to acute respiratory alkalosis (Ge et al., 2006). Similar results were also reported by Gledhill et al. (1975), where participants were exposed to normoxic and hypoxic conditions during coached hyperventilation (respiratory alkalosis) for 26 h to assess renal responses. In both hypocapnic and hypoxic conditions, urine pH became alkalinized at 6 h of hypocapnia (i.e., simulated high altitude‐mediated hypocapnia). Given our urine pH measures were aerobic, it is likely that CO_2_ in urine samples immediately equilibrated with ambient air upon obtaining the sample. This is supported by the fact that the hypocapnia associated with ascent did not affect urine pH, as hyperventilation‐induced hypocapnia did in Ge et al. (2006) and Gledhill et al. (1975), where anaerobic urine samples were assessed.

However, in our study, oral acetazolamide administration did alkalinize urine pH relative to baseline at all altitudes, both acutely and chronically with ascent to high altitude (see Table 3 and Figure 9a, b). This is consistent with a study by Galdston (1955), where patients who were administered oral acetazolamide had an increase in urine pH compared to baseline. The increased urine alkalinity in our Az group suggests that systemic H^+^ retention facilitated by acetazolamide was detected in our aerobic urine samples. On balance, although aerobic urine pH measures may not have utility in assessing renal responses to altitude‐mediated respiratory alkalosis, it does appear to have utility in assessing binary acetazolamide status in large groups of trekkers at any altitude.

### 4.8 Conclusions

We made physiological measures during incremental ascent to HA in the Nepal Himalaya to 5160 m with a total of 46 participants. To quantify VA, we used portable devices to assess $P_{\mathrm{ETC}O_{2}}$ and $S_{pO_{2}}$, which were then plotted on modified Fenn diagrams. After calculating the AUC on these individually constructed diagrams, they were distributed into low, moderate and high degree of acclimatization cohorts. We report normative values for VA for participants with varying degrees of acclimatization and compared our values with those calculated from previously published studies. Second, we also found that a separate group of participants self‐administering a prophylactic dose of acetazolamide (250 mg BID; *n* = 20) with ascent had significantly smaller AUCs on modified Fenn diagrams during incremental ascent to 5160 m compared to the acetazolamide‐free groups, suggesting a higher magnitude of ventilatory acclimatization. We suggest that using this method of assessing VA during incremental ascent using AUC on individually constructed modified Fenn diagrams may be applied to large trekking groups during HA expeditions, because of its portability and congruency with known physiology, although this novel analytical method requires further validation in controlled experiments.

## AUTHOR CONTRIBUTIONS

Conception and design of the work: Ken D. O'Halloran, Thomas D. Brutsaert, Mingma T. Sherpa, Trevor A. Day; acquisition, analysis, or interpretation of data for the work: all authors; drafting the work or revising it critically for important intellectual content: all authors. In addition, all authors, approved the final version of the manuscript, agree to be accountable for all aspects of the work in ensuring that questions related to the accuracy or integrity of any part of the work are appropriately investigated and resolved. All persons designated as authors qualify for authorship, and all those who qualify for authorship are listed.

## CONFLICT OF INTEREST

The authors declare no conflicts of interest.

## FUNDING INFORMATION

Financial support for this work was provided by a Natural Sciences and Engineering Research Council of Canada Discovery Grant (RGPIN‐2016‐04915). J.K.L. was funded by the Department of Physiology, University College Cork, Ireland.
